# Investigation of the First African Swine Fever Outbreak in a Domestic Pig Farm in Hong Kong

**DOI:** 10.1155/2023/1720474

**Published:** 2023-05-18

**Authors:** Yun Young Go, Jeremy H. P. Ho, Karina W. S. Tam, Maedeh Kamali, Yiwen Zhang, Candy C. Y. Lau, Song Hao Li, Michael T. Wilson, Zhihao Guo, Runsheng Li, Guoqian Gu, May P. Y. Tse, Fraser I. Hill, Carrie Batten, Amanda Corla, John Flannery, Anne Conan, Christopher J. Brackman, Dirk U. Pfeiffer

**Affiliations:** ^1^Department of Infectious Diseases and Public Health, Jockey Club College of Veterinary Medicine and Life Sciences, City University of Hong Kong, Hong Kong SAR, China; ^2^Agriculture, Fisheries and Conservation Department, Government of the Hong Kong Special Administrative Region, Hong Kong SAR, China; ^3^Centre for Applied One Health Research and Policy Advice, City University of Hong Kong, Hong Kong SAR, China; ^4^CityU Veterinary Diagnostic Laboratory, City University of Hong Kong, Hong Kong SAR, China; ^5^The Pirbright Institute, Ash Road, Woking, Surrey, UK

## Abstract

In this study, we describe the epidemiological investigation of the first African swine fever (ASF) outbreak in a local domestic pig farm in the New Territories of Hong Kong in 2021. In the outbreak farm, several affected pigs presented clinical and pathological signs consistent with ASF, while the remaining pigs showed nonspecific clinical signs or did not exhibit any clinical signs. The relative low morbidity and mortality of ASF on this farm resulted in delayed detection and implementation of the control response. Despite this delay, no further spread of the disease from this farm to other farms or wild boars was observed. The clinical presentation of ASF in terms of morbidity and mortality on this farm indicated that it is essential for effective surveillance aimed at early detection for farmers, veterinarians, and pathologists to be educated about the different ways ASF can express itself in domestic pig populations. Epidemiological investigations consisted of field inspection, interviews with farm personnel to assess the management and biosecurity practices within the farm, and laboratory testing of animal and environmental samples. In addition, the complete genome of ASFV was obtained directly from the tissues of an infected pig to facilitate the epidemiological investigation. The genetic relationship at the whole genome level indicated that the isolate shared the highest level of similarity with genotype II ASFVs, including a 2019 isolate from Guangdong province, China (GD2019). Overall, the information presented here from the on-farm investigation with that from diagnostic testing and molecular analyses provides a basis for informed actions to prevent future incidents in farms with similar characteristics. Furthermore, this study highlighted the need to increase current knowledge about the molecular diversity amongst circulating viruses and potentially trace the source of infection.

## 1. Introduction

African swine fever (ASF) is a contagious hemorrhagic viral disease affecting domestic pigs and wild boars [1]. The disease is caused by African swine fever virus (ASFV), and it is on the list of notifiable diseases of the World Organisation for Animal Health (WOAH) due to its high mortality rate and significant socioeconomic impact on pork production and trade in affected countries [2–5]. Morbidity and mortality for ASF can reach up to 100% in naïve domestic pig populations [6]. The clinical signs can vary in severity, from acute to chronic disease, depending on the complex interactions between virus and host factors. Peracute and acute presentations are the most common forms of the disease, characterized by rapid disease progression, with high fever, lethargy, anorexia, and respiratory distress [7, 8]. The acute form typically occurs in naïve animals, often presenting with clinical signs of skin hemorrhages and cyanosis, particularly on body extremities, along with signs of the peracute form [7, 9]. At present, disease control relies on rapid diagnosis, restriction of animal movement, control of trade in pork products, and culling of infected and in-contact animals. ASFV is transmitted between pigs via multiple pathways, including direct contact with infected pigs, consumption of contaminated food products, or fomites such as vehicles, workers, and equipment. ASFV can persist for extended periods of time in contaminated products or vehicles, thus enabling spread to places far from the original infected premises via indirect contact, making disease control more challenging [10]. In addition, clinical signs often resemble those of other common swine diseases. Therefore, the initial cases of ASF may be mistakenly attributed to endemic diseases in the farm or region causing delay in diagnosis and control.

African swine fever virus is a DNA arbovirus and the sole member of the genus *Asfivirus* within the *Asfarviridae* family [11]. It is an enveloped virus with a large double-stranded DNA genome of 170 to 193 kb in size with terminal inverted repeats and hairpin loops [12]. Depending on the virus strains, the genome has a conserved central region of about 125 kb with 5′- and 3′-termini of variable sizes and it encodes 150 to 167 open reading frames [6]. Currently, 24 different genotypes of ASFV have been identified based on the B646L gene, which encodes the capsid protein p72 [13, 14]. The occurrence of ASF was mainly limited to African countries until the virus was introduced to Georgia from eastern Africa in 2007, after which it widely spread across Europe. In 2018, Georgia-07-like genotype II ASFV was introduced to China via Russia, and it rapidly spread to most Chinese provinces causing unprecedented disaster and challenges to the world's biggest pork industry [15–17]. Subsequently, ASFV reached many other Asian countries, including Vietnam, Cambodia, Laos, the Philippines, Myanmar, Indonesia, South Korea, Mongolia, Timor-Leste, Papua New Guinea, and India [15, 18–21]. Recently, the emergence of genotype I ASFV and genetically diverse ASFV strains carrying significant variations and/or deletion patterns of the genome causing attenuated phenotype has been reported in China [22]. Unfortunately, the emergence of genotype I and II variants with milder disease phenotype and lower mortality than the initial strain [22, 23] makes an early diagnosis of ASF and epidemiological investigations even more challenging.

Despite the widespread occurrence of ASFV worldwide, only a limited number of outbreak investigations have been published in the scientific literature [24]. Thus, more knowledge needs to be generated from epidemiological outbreak investigations that will inform actions on farms to prevent the introduction and limit the spread of the virus within and between farms. This study reports the epidemiological investigation of the first African swine fever outbreak detected on a local domestic pig farm in the New Territories of the Hong Kong Special Administrative Region (HK SAR) in early 2021. Immediate action and notification to WOAH were conducted; however, there has been a significant lag in our report progress due to the COVID-19 pandemic that impacted the territory. Although ASFV has been present in Mainland China since August 2018, the virus was not detected in Hong Kong domestic pig farms and wild boar until the outbreak, which is being presented here. Investigations were conducted on the outbreak farm to understand the extent of the spread of ASFV within the farm and to explore possible routes of ASFV introduction. It consisted of field inspection, interviews with farm personnel, and laboratory testing of environmental samples to assess the management and biosecurity practices within the farm. The information generated by the on-farm investigation, diagnostic testing, and molecular analyses is described to provide a basis for informed actions on farms as well as a better understanding of the outbreak, which may contribute to preventing future incidents in farms with similar characteristics. Furthermore, this study highlighted the need to analyze additional ASFV genomes from the region and beyond to increase current knowledge about the molecular diversity amongst circulating viruses and potentially trace the source of infection.

## 2. Materials and Methods

### 2.1. Farm Investigation Study

The disease investigation by the City University of Hong Kong (CityU) Veterinary Service began upon notification by the farm owner (farmer) of a suspected disease outbreak with a sudden increase in deaths of 6.5 months old finisher pigs on Jan 29, 2021 (Day 0). During the initial disease investigation, a dead pig was sent to CityU Veterinary Diagnostic Laboratory (VDL) for postmortem examination and diagnostic testing. After suspecting an ASFV infection in the farm, the spleen and lymph nodes from the index pig were collected and submitted to the Tai Lung Veterinary Laboratory (TLVL) of the Agriculture, Fisheries, and Conservation Department (AFCD) of HK SAR for laboratory testing. Once ASFV infection was confirmed by PCR, a further outbreak investigation was carried out by the AFCD. The farm investigation was conducted with reference to the procedures described in the Food and Agriculture Organisation Animal Production and Health Manual on African swine fever for veterinarians [25]. It consisted of field inspection, interviews with farm personnel, and laboratory testing of environmental samples to assess the management and biosecurity practices within the farm and the potential routes of ASFV introduction. In addition, apart from the affected farm, the AFCD performed surveys to monitor pig health and biosecurity measures in the remaining 42 local domestic pig farms in HK SAR via questionnaires and farm visits. The questionnaires aimed to collect the health information of pigs, including abnormal death, relevant clinical signs for early detection of suspected ASF cases, and biosecurity measures implemented on the farm. The farmers were also requested to submit nasal swab samples of sick and/or dead pigs for ASFV PCR testing.

The affected pig farm, managed by the farmer and several farm workers, was located close to a residential village and a main road of the New Territories of HK SAR. Three other domestic pig farms were located approximately 0.2 km, 1.3 km, and 1.5 km from the index pig farm. An animal carcass collection site, where animal carcasses from nearby livestock farms would be collected and disposed of at a landfill, was around 1.1 km from the index pig farm. No abattoirs were located within 3 km of the farm. The farm consisted of 20 sheds distributed over eight buildings, keeping a total of 4,000 pigs, approximately. In terms of farm management, the farm had implemented a number of biosecurity measures, which were generally in line with those in the guidelines and educational seminars provided by the AFCD, and inspected from time to time during farm visits. Pigs of different production stages, including breeders, finishers, growers, nursery pigs, and suckling piglets, were housed in separate sheds and managed by dedicated farm workers who would not handle pigs of other production stages to prevent cross-contamination. A vehicle disinfection wheel bath was present at the farm entrance, where any vehicles entering the farm were subjected to disinfection. In addition, disinfection footbaths were available at the entrance of the pig sheds. The animal loading/unloading platform was located outside the production area at the farm entrance, where pig transport vehicles could load pigs outside the farm entrance without entering the production area. Unauthorized entry of vehicles and personnel into the farm was prohibited. Outside the production area, there were living areas, such as living and dining rooms, where farm workers shared common areas. Every farm worker was also provided with a dormitory next to the farm, while the farmer lived next to the farm in a separate building.

### 2.2. Sample Collection

During the investigation, a risk-based sampling approach was adopted to increase the probability of sampling ASFV-infected pigs. At least one pig was selected for a blood sample collection from each of the 20 sheds on the farm based on at least one of the following purposive sample selection criteria, in order of priority: (i) showing relevant clinical signs (e.g., erythema and nasal discharge), (ii) being weak (e.g., recumbent, depressed, and dog-sitting position), or (iii) from locations within the shed considered at higher risk of infection (e.g., locations nearby the entrance of a shed or pigs with frequent interactions with farm workers such as for artificial insemination). Blood samples were collected with sterile syringes and stored in EDTA vacutainer. Forty-five environmental swab samples were collected from different sheds and areas of the farm and placed in a viral transport medium (medium 199 supplemented with 0.5% bovine serum albumin and antibiotics) for temporary storage before submission to the TLVL. In addition, six feed samples were collected from the farm in a sterile plastic container.

In addition, tissue samples from pigs with and without clinical signs were collected during the culling operation. In brief, pigs were culled using carbon dioxide in enclosed chambers in the following order: (1) pigs in the index shed, (2) breeders, (3) finishers, (4) growers, (5) nursery pigs, and (6) piglets. The spleen, lymph nodes, and/or blood samples were collected from pigs presenting clinical signs suggestive of ASFV infection during the culling process. Pigs without apparent clinical signs were also selected for sampling on a convenience basis to determine the extent of the outbreak within the farm. The collected tissue samples were placed in a viral transport medium and transported to the TLVL for ASFV testing.

### 2.3. Postmortem Examination

As a standard procedure, a postmortem examination was conducted by CityU VDL. Tissue samples were collected, including lung, small intestine, large intestine, kidney, liver, synovium of the hock, spleen, brain, spinal cord, tonsil, pancreas, skin, and renal lymph nodes in 10% neutral buffered formalin and processed for a histopathology examination. Thin tissue sections (5 *μ*m) were stained with hematoxylin and eosin (H&E) and a detailed histopathological evaluation was conducted by light microscopy. In addition, tissue samples, such as lung, small and large intestines, synovium, meninges, kidney, liver, and lymph nodes, were collected and subjected to molecular diagnostic tests. The laboratory diagnostic tests included a panel of bacterial and viral pathogens commonly found in the region (Supplementary Table S1). Spleen and lymph node samples were stored at −80°C and subsequently sent to TLVL of the AFCD for ASFV diagnostic tests.

### 2.4. Real-Time PCR and Molecular Genotyping of ASFV

DNA was extracted from blood and homogenized spleen and lymph node samples using the NucliSens easyMAG extraction kit (BioMerieux, Marcy l'Etoile, France) following the manufacturer's instructions. For initial diagnosis, the primer set (forward: 5′-CTGCTCATGGTATCAATCTTATCGA-3′ and reverse: 5′-GATACCACAAGATCRGCCGT-3′) and probe (5′-FAM-CCACGGGAGGAATACCAACCCAGTG-BHQ1-3′) were used for detection of ASFV p72 gene by real-time PCR, as previously described [26]. For Sanger sequencing, 257 bp fragments corresponding to a region of the p72 gene were amplified using primers (forward: 5′-AGTTATGGGAAACCCGACCC-3′ and reverse: 5′-CCCTGAATCGGAGCATCCT-3′), as previously described [27]. The PCR amplicons were purified using a QIAquick PCR Purification kit (Qiagen, Germany).

### 2.5. ASF Haemadsorption (HAD) and Virus Isolation (VI)

Four selected samples (lymph node, spleen, and two EDTA blood samples) were tested by haemadsorption (HAD) and virus isolation (VI) at the WOAH reference laboratory for ASFV at The Pirbright Institute, United Kingdom. ASFV HAD titrations were performed using porcine bone marrow cells (PBMs) extracted from the long leg bones of 4-week-old uninfected pigs. The PBMs were seeded in 96-well plates at a density of 1.0–1.6 × 10^7^ cells/ml in Earle's Balanced Salt Solution (EBSS) supplemented with 10% heat-inactivated porcine serum, 100 U/ml penicillin, 100 *µ*g/ml streptomycin, and 1% HEPES solution, incubated in a humidified chamber at 37°C with 5% CO_2_ for 3 days. The homogenized tissue (*n* = 2) and EDTA blood samples (*n* = 2) underwent a series of ten-fold dilutions to inoculate the PBMs in freshly prepared EBSS containing 15% heat-inactivated porcine serum, 100 U/ml penicillin, and 100 *µ*g/ml streptomycin. Samples were run in quadruplicates, and ASFV Malta 78 isolate was used as a positive control. Plates were incubated for 6 days, and results were calculated using the Spearman–Karber formula to express titers as log_10_ HAD_50_/ml. HAD-positive wells were pooled and centrifuged at 1500 × *g* for 5 minutes. The supernatant was used to inoculate fresh PBMs in culture flasks containing EBSS with 15% heat-inactivated porcine serum, 100 U/ml penicillin, and 100 *µ*g/ml streptomycin. Inoculated PBMs were incubated in a humidified chamber at 37°C with 5% CO_2_ and harvested after 3 days once HAD was observed. Viral propagation was confirmed on the cell culture supernatant by performing ASFV real-time PCR described above.

### 2.6. Complete Genome Sequencing and Analysis

Total DNA was purified from the homogenized spleen tissue from the index pig using a DNeasy® Blood and Tissue kit (Qiagen, Germany) according to the manufacturer's instructions. The quality and quantity of DNA were determined using a NanoDrop spectrophotometer (ThermoFisher, USA). To directly sequence the full-length viral genome from clinical samples of the infected pig, whole-genome sequencing (WGS) was performed using both nanopore long-read sequencing (LRS) and Illumina short-read sequencing (SRS) techniques. In brief, 4 *µ*g of total DNA was used for the MinION nanopore library preparation using Ligation Sequencing Kit (LSK110) (Oxford Nanopore Technologies (ONT)) according to the manufacturer's instructions. Sequencing was performed in a MinION sequencer (MIN-101B) with R9.4.1 flow cells (FLO-MIN106D). The DNA base calling was performed using Nanopore Guppy (v 5.0.11; ONT) with a high-accuracy model (dna_r9.4.1_450 bps_sup.cfg). The viral genome was also sequenced on an Illumina® Novaseq 6000 with paired-end mode according to the standard protocols of Novogen Co., Ltd. (Beijing, China).

Minimap2 v2.17-r941 [28] with default parameters for Nanopore reads (-ax map-ont) was used to align the long reads to the cross-references containing 127 full-length ASFV genomes, and the viral reads were extracted using SAMtools v1.9 [29]. Then, de novo assembly was performed with parameters specifying the estimated genome size (200 kbp) and Nanopore raw reads using Canu v 2.2 [30]. The resulting assembly of a single contig consisting of 190 kb was polished twice with Racon v 1.4.3 [31] using Nanopore reads and six times with Illumina reads using Pilon v 1.24 [32]. Finally, the draft genome annotation was performed by liftover annotation from the nearest known strain, ASFV Estonia 2014 (GenBank LS478113), using RaGOO v 1.11 [33].

### 2.7. Conventional PCR and Sanger Sequencing

Sequence regions with ambiguous reads, mainly nucleotide insertions/deletions (indels) present in homopolymer tracts of the assembled reads from LRS and SRS, were subjected to Sanger sequencing for further confirmation. The amplification of gene fragments was performed using Platinum™ Taq DNA Polymerase High Fidelity (ThermoFisher, USA) and the following PCR conditions: initial denaturation at 94°C for 2 min followed by 35 cycles each of 94°C for 15 sec, appropriate annealing temperature for each primer set for 30 sec, 68°C for 1 min. Subsequently, PCR amplicons were resolved by electrophoresis and purified using a QIAquick gel extraction kit (Qiagen) following the manufacturer's instructions. Sanger sequencing was done through a commercial company (BGI Genomics, Hong Kong). Sequence data were analyzed using CodonCode Aligner version 9.0.1 (CodonCode, USA) and Geneious Prime® 2020.2.5 (Biomatters, Ltd., New Zealand).

### 2.8. Phylogenetic Analysis

Pairwise genome sequence alignment was performed using Geneious Prime® 2020.2.5. Multiple nucleotide alignment was conducted using MAFFT v7.475 [34]. The phylogenetic trees were built using the maximum likelihood method with 1,000 bootstraps using RaxMLVersion 8 [35]. For the input sequences of the p72 and CD2v trees, all available full-length sequences for p72 and CD2v were directly downloaded from GenBank. Identical sequences were merged before alignment, resulting in 53 nonredundant sequences (out of 203) for p72 and 62 nonredundant sequences (out of 352) for CD2v. A total of 127 nonduplicated whole-genome sequences were used for the whole-genome analysis. The ORFs from 121 conserved orthologs, which contained at least >80% alignable region with the ORFs from HK202103 strain, were used in the concatenated sequence for alignment. The visualization of the tree was carried out using ETE 3 [36].

### 2.9. Data Availability

The full-length genome sequence of ASFV HK202103 has been deposited in the GenBank database under the accession number OK358852.

## 3. Results

### 3.1. Outbreak Characteristics

The farm is located in a rural area and is next to several industrial warehouses. The road directly outside of the farm entrance connects to a main road around 300 meters away, and it also connects to some organic crop farms on the other side, which are about 300–400 meters away. The outbreak investigation started on Jan 29, 2021 (Day 0), when the farmer notified the CityU Veterinary Service of an unusual increase in deaths of finisher pigs. As recalled by the farmer, the disease onset was first noticed on Jan 24, 2021, involving initially only one pen in the index shed, with less than 10% of pigs showing nonspecific clinical signs of inappetence. However, despite the administration of antibiotics, there was increased morbidity of pigs with clinical signs such as fever, red and purple discoloration of extremities (e.g., ears), respiratory symptoms, and subsequent mortality. In addition, similar clinical signs were manifested in pigs from other pens in the same shed. In response, the farmer removed those affected pigs showing relevant clinical signs from the original pens to an isolation pen near the shed entrance. To prevent further spread of the disease within the farm, the farmer also implemented additional biosecurity measures, such as not sharing equipment between sheds and wearing personal protective equipment (disposable coveralls, paper caps, facemasks, gloves, and rubber boots) inside the index shed.

**Figure 1:**
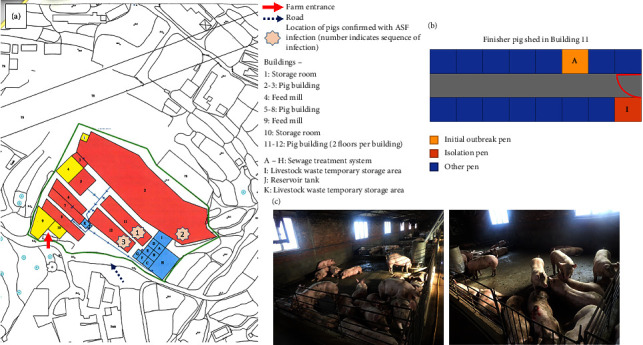
Map of the farm buildings and floor plan of Building #11 where the initial outbreak was detected. (a) The map of the farm and its surroundings are depicted. The sheds where the ASF-related incidents occurred are indicated in the sequence of events by stars. (b) The floor plan of the finisher pig “index” shed in Building #11, containing 16 pens, is depicted. (c) Health conditions of pigs housed in Building #11. A few pigs presented mild skin reddening and nasal discharge on Day 5.

**Figure 2:**
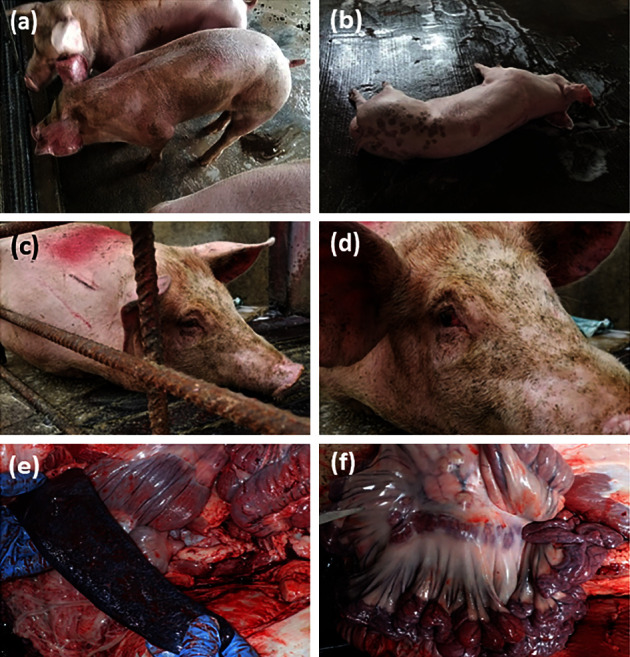
Clinical signs and gross lesions of pigs suspected of ASFV infection. On Day 7, (a) pigs with diffuse reddening of pinnae and (b) a dead pig with purplish-blue discoloration on the tips of the pinnae were found in Building #11. On Day 13, (c) a recumbent pig with diffuse reddening of pinnae and (d) conjunctivitis was found in Building #12. Gross lesions, including (e) mild splenomegaly (round edges of the spleen) with congestion and (f) mesenteric lymph node enlargement and hemorrhages, are shown.

**Figure 3:**
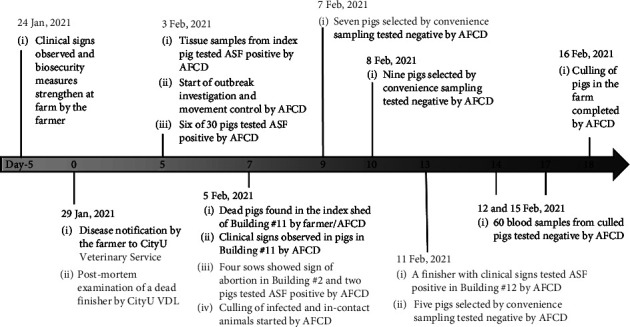
Timeline of the ASF events and outbreak investigation in the domestic pig farm.

A shed located at the upper level of Building #11, illustrated in Figure 1(a), was identified as the “index shed,” which consisted of 16 pens with eight on each side separated by a central corridor housing 245 finishers of around 6.5 months old when the suspected outbreak was noted (Figure 1(b)). The pens on each side were separated by fences made of metal bars through which direct contact between pigs in adjacent pens was possible. In the index shed, clinical signs were first noticed in pigs from pen A, including the pig that was later found dead (index pig) by the farmer and submitted to CityU VDL for postmortem examination and laboratory testing on Day 0, followed by those in pen I (Figure 1(b)). The two affected pens (Pen A and I) held 38 pigs in total that exhibited 63% morbidity (24/38) and 11% mortality (4/38) during the five days prior to the first farm visit associated with the outbreak. The affected pigs displayed inappetence, fever, recumbency, nasal discharge, and purplish-blue discoloration of extremities (Figure 1(c)). On Days 1 and 2, the farmer isolated clinically affected pigs and selectively culled pigs showing relevant clinical signs in the index shed, totaling around 40 pigs, as a preventive measure. On Day 5 (Feb 3, 2021), soon after the laboratory confirmation of ASFV from the index pig, the AFCD issued a legal order to suspend the in-and-out movement of pigs and materials from the farm. In addition, all 20 sheds on the farm were inspected in a one-way movement direction, with the index shed being the last to be inspected to minimize the risk of further spread of ASFV within the farm. Blood samples from nine pigs (*n* = 9) were collected from the index shed during the inspection. The pigs were selected from nine different pens based on the manifestation of relevant clinical signs, such as mild skin reddening and nasal discharge. Most of the remaining pigs from the index and other sheds showed no apparent clinical signs. In addition, 21 pigs from the remaining sheds without disease manifestation were sampled for laboratory testing, resulting in sample collection from 30 pigs. Six of nine pigs from the index shed tested ASFV positive by qPCR with Ct values ranging from 15.3 to 22.7, whereas the remaining 24 samples were negative for ASFV. On Day 7, several more pigs were found dead, and the clinical signs, such as reddening or purplish-blue discoloration of extremities, became more prominent amongst affected pigs in the index shed (Figures 2(a) and 2(b)). In addition, four sows adjacent to each other in a breeder shed in Building #2 presented clinical signs of inappetence and abortion. Blood samples from the sows were collected and submitted for laboratory tests. The qPCR results confirmed two of the four sows as ASFV positive with Ct values of 16.3 and 17.0. On Day 13, a finisher pig in the upper-level shed of Building #12 showed clinical signs of inappetence, vomiting, recumbency, conjunctivitis, and diffused reddening of ear pinnae (Figures 2(c) and 2(d)). The finisher was subjected to an on-site postmortem examination that revealed gross findings of mild splenomegaly with congestion and mesenteric lymph node enlargement with hemorrhages (Figures 2(e) and 2(f)). The lymph node and spleen samples collected during the necropsy were tested ASFV positive by qPCR with Ct values 21.4 and 16.5, respectively. Ten pigs from three buildings (two sows from Building #2, seven pigs from Building #11, and one finisher from Building #12) were tested ASFV positive by qPCR. Among ASFV-positive samples, the virus was successfully isolated from three pigs and demonstrated to be haemadsorbing (Table 1). Apart from the specific pigs and samples mentioned above, another 21 pigs, around one-third with obvious clinical signs such as reddening of the skin and ear pinnae, were necropsied during the culling operation, which started on Day 7 and was completed by Day 18. These 21 pigs included seven pigs sampled on Day 9 (Feb 7, 2021), nine pigs on Day 10 (Feb 8, 2021), and five pigs on Day 13 (Feb 11, 2021). There were no significant internal gross findings, and these pigs tested ASFV negative. In addition, blood samples were collected from another 60 culled pigs, of which 30 clotted blood and 30 EDTA blood samples were collected on Day 14 (Feb 12, 2021) and Day 17 (Feb 15, 2021), respectively, on a convenience basis during culling. The results were all negative for ASFV on qPCR. The detailed timeline of the outbreak investigation, sampling for laboratory tests, and outcomes are summarized in Table 1 and Figure 3. In addition, the six feed samples and 45 environmental swab samples collected during the field investigation all tested negative for ASFV on qPCR.

### 3.2. Postmortem Examination and Laboratory Testing of the Index Pig

**Figure 4:**
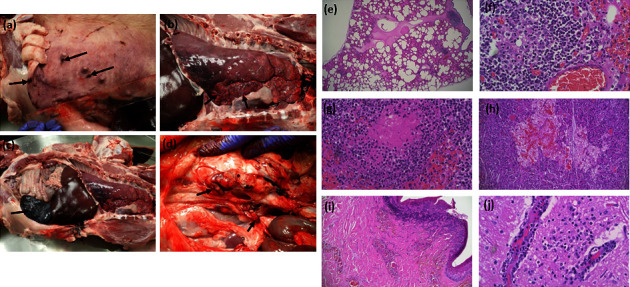
Post-mortem examination of index ASFV infected pig. Left panels: gross findings of index pig on day 0. (a) Skin lesions of multiple well-demarcated irregular patches (black arrows) are slightly raised and often with a rim of red to brown discoloration. (b) Pulmonary congestion and diffuse pulmonary edema (red arrows: prominent interlobular septa). (c) Splenomegaly with mildly round edges and congestion (red arrow). (d) Renal lymph node hemorrhages. Right panels: histopathological examination of index pig on day 0. (e) Multifocal to coalescing edema in the alveolar spaces and the interlobular septa (lung, H&E, 40X). (f) Bronchus-associated lymphoid tissue necrosis and fibrinonecrotic alveolitis/vasculitis (lung, H&E, 400X). (g) Lymphoid necrosis (spleen, H&E, 400X). (h) Necrotic focus with hemorrhage (pancreas, H&E, 100X). (i) Necrotic epidermis and suppurative infiltrate with fibrin thrombi in the blood vessels in the superficial dermis (skin, H&E, 100X). (j) Vasculitis and spilling of inflammatory cells to the adjacent cerebral parenchyma (brain, H&E, 400X).

The index pig, a female Landrace–Yorkshire–Duroc cross finisher, was subjected to tissue sample collection and postmortem examination at the CityU VDL on Day 0. The postmortem examination revealed gross findings, including bilateral red discoloration of the skin of the ear pinnae and multiple, well-demarcated, irregular patches of red to brown skin discoloration, predominantly in the ventrum (Figure 4(a) (A)). The trachea was filled with froth, and the lungs were diffusely heavy and wet, interpreted as acute pulmonary edema and congestion (Figure 4(a) (B)). Splenomegaly was noted (Figure 4(a) (C)). The renal and right parotid lymph nodes showed moderate to multifocal hemorrhages (Figure 4(a) (D)). Histopathology examination found acute pulmonary edema, necrosis in the lung, small and large intestines, mesenteric and renal lymph nodes, kidney, liver, synovium of the hock, spleen, tonsil, and pancreas, along with frequent lymphocyte necrosis in lymphoid tissue. Fibrinonecrotic vasculitis, fibrin thrombi, and hemorrhages were found in the lungs, spleen, pancreas, and skin (Figures 4(b) (A)–(E)). Vasculitis and meningoencephalomyelitis were noted in the brain and the spinal cord to a lesser extent (Figure 4(b) (F)). The histopathological changes in the tissue sections were consistent with DIC and are summarized in Supplementary Table S2. Tissue samples were subjected to laboratory tests for common and endemic swine viral and bacterial diseases, among which only porcine circovirus type 2 and *Haemophilus parasuis* tested positive (Supplementary Table S1). Given the negative diagnostic results of endemic diseases commonly found in local pig farms with similar clinical signs and necropsy findings (i.e., porcine reproduction and respiratory syndrome), a likelihood of ASFV infection was suspected. Therefore, relevant tissue samples collected from the index pig during postmortem examination were submitted to the TLVL of AFCD for ASFV testing, where lymph nodes and spleen were confirmed positive for the virus by qPCR with Ct values of 20.5 and 18.2, respectively, on Day 5 (Feb 3, 2021). On Mar 22, 2021, ASFV was successfully isolated from these tissue samples, and the virus tested positive for HAD by the Pirbright Institute, United Kingdom, as summarized in Table 1.

### 3.3. Farm Investigation

The outbreak investigation consisted of field inspection, interviews with farm personnel, and laboratory testing of environmental samples to assess the management and biosecurity practices within the farm. During the interview, the farmer confirmed that swill feeding was prohibited on the farm. In addition, since the farm workers were provided with food by the farm management, the likelihood of farm workers bringing pork or pork products to the farm was very low although bringing their own food was still possible. Given the evidence that swine feed and/or ingredients can be potential vectors for ASFV transmission, the feed products were also investigated. Pigs were fed with dry mixes made with feed products from different origins. The feed was stored at room temperature in an open but dedicated storage area (Building #4 in Figure 1), which appeared neat and clean. Interestingly, the farmer recalled a recent change in the package of a feed product (phosphate) and the supplier of wheat powder, which was suspected as a potential source of ASFV introduction. Thus, feed products containing phosphate and wheat powder were tested; however, no ASFV genome was detected from any samples indicating that feed may not be the source of ASFV introduction. Moreover, the farmer and workers reported no recent changes in terms of the drugs and vaccines used on the farm. They also emphasized that no ASF vaccines had been used on the farm or found during the investigation. Hence, the outbreak was unlikely related to using unlicensed ASF vaccines.

Barriers at the outer boundary of the premises, composed of concrete and brick walls of around two meters in height with a solid metal gate, were considered adequate for preventing wild boar intrusion. In addition, the fact that domestic pigs were kept inside individual buildings would have further reduced the likelihood of potential contact between domestic pigs and wild boars. The last occasion of introducing new pigs into the index farm was on Apr 11, 2019, when 56 gilts and four boars were imported from Taiwan. They all passed quarantine with satisfactory inspection outcomes on May 22nd, 2019. Considering that the last introduction of pigs to the farm before the ASF outbreak was over 21 months from a place that was ASF-free at the time, and no other outbreaks were detected in local farms over the period, the introduction of new pigs was unlikely to be the source of the outbreak.

Possible fomite transmission via contaminated materials, pig transport vehicles, or personnel was also investigated as a potential source of the outbreak. The farmer indicated that each production stage of pigs was taken care of by dedicated farm workers who would not handle pigs of other production stages to prevent cross-contamination. However, there were common living areas where the farm workers gathered to dine or watch TV. In addition, farm workers infrequently changed clothes or footwear when moving between the production and living area. Environmental swab samples were collected from the farm workers' common living areas, clothes, shoes, floor surfaces, and dormitory stairs and tested to determine any potential contamination with the virus. However, no ASFV genome was detected from the collected samples. During the interview, the farmer indicated that his father also owns a pig farm in Mainland China, which previously had an ASF outbreak. His father's last visit to the index farm was in Jan 2020, i.e., over one year before the outbreak. At that time, he took appropriate disinfection and cleaning procedure (i.e., showering) before entering the farm. He also claimed there was no exchange of materials with his father's farm in Mainland China. Neither the farmer nor other farm workers had been back to Mainland China since early 2020 due to COVID-19 travel restrictions in both HK SAR and Mainland China during the pandemic. Nevertheless, despite the farmer's claims, it remained uncertain whether there had been any indirect material transfer between the farmer and his father or whether the workers unintentionally brought anything to the premises.

Regarding biosecurity measures for vehicles, all cars were cleaned before entering the farm, including disinfection of wheels and surfaces. The farm entrance was equipped with a high-pressure water hose and disinfectant spray. Disinfection wheel bath for cars was also provided inside and near the farm entrance, filled with Virkon S (1 : 100) disinfectant. Only three vehicles for transporting feed would visit and enter the farm on an ad hoc basis. These vehicles also visited other pig farms to deliver fish meals (originating from Denmark), feed premix (originating from Belgium), and wheat powder (originating from Mainland China). Since ASFV was not detected in other farms, it is unlikely that the feed vehicles were the source of virus introduction to the farm. Vehicles transporting pigs between pig farms and abattoirs visited the index farm almost every day but did not enter the production area of the farm. The loading and unloading of pigs were performed in the pig loading platform outside the farm entrance. Given that most live pigs entering the local abattoirs are imported from Mainland China, it was reasonable to assume that abattoirs in the region could be the potential routes of ASFV entry. Moreover, the farmer recalled that the farm worker in charge of the index shed was also responsible for frequent carcass disposal for the farm. Thus, it is plausible that the animal carcass collection site and the relevant vehicles could have served as a potential route of disease entry through contamination of the concerned farm worker resulting in the eventual ASF outbreak.

### 3.4. Characterization of the Complete Genome Sequence of ASFV HK202103

A complete genome sequence of ASFV isolated from the spleen tissue of the index pig found dead on Day 0 was generated using Nanopore LRS and Illumina SRS techniques. Direct viral genome DNA sequencing using the Nanopore platform generated 5,596 viral reads with a total yield of approximately 20 Mbp (about 100X for the ASFV genome) with a maximum length of 67 kbp. Approximately 22G raw sequence reads were generated, including the host and viral genomic DNA. The average Phred quality scores for raw Nanopore reads were around 14. The final assembly was polished twice using Nanopore reads and six times with NGS reads, which resulted in a single contig of 192,298 bp in length. The complete genome designated ASFV HK202103 consisted of 196 predicted protein-coding genes, including MGF 100 (3 members), MGF 110 (13 members), MGF 300 (3 members), MGF 360 (19 members), and MGF 505 (10 members). Analysis of p72 and CD2v sequences indicated that the virus was within ASFV genotype II, as shown in Supplementary Figures S1 and S2. The B646L gene sequence, encoding the major capsid p72 protein, showed 100% nucleotide identity with Georgia 2007/1 and 46 other isolates, including those identified from Eastern Europe (Estonia 2014, CzechRepublic 2017/1, HU_2018), China (AnhuiXCGQ, Pig/HLJ/2018, Wuhan 2019-1 and 2, and China/GD/2019), and Vietnam (VNUA/TB-ASF1, VNUA HY-ASF1, and NgheAn 2019) (Supplementary Table S3). Correspondingly, there were no deletions or mutations in the EP402R gene of ASFV HK202103 encoding the CD2v protein, which showed 100% nucleotide identity with those of ASFV-SY18 (MH766894.1) and HuB20 (MW521382) from Mainland China, as well as ASFV_Hanoi_2019 (MT166692) from Vietnam, denoting that HK202103 belongs to CD2v serogroup 8 **(**Supplementary Figure S2**)**. The result was consistent with the positive HAD phenotype confirmed by HAD assay (Table 1). In addition, the HK202103 strain had an additional tandem repeat sequence (5′-GGAATATATA-3′) between the I73R and I329L, characteristic of the intergenic region (IGR) II variant of the genotype II group. Further analysis of genetic markers such as the central variable region (CVR) of ORF B602L and the IGR of MGF 505 9R/10R indicated that HK202103 belongs to CVR variant 1 and subgroup MGF-1, respectively.

Comparative analysis of the complete genome indicated that the overall nucleotide sequence similarity of ASFV HK202103 was 99.754%, with an additional 1,019 bp at the 5′-end and a 955 bp longer tail at the 3′-end compared to genotype II reference Georgia 2007/1 genome. In addition, a pairwise comparison between HK202103 and Georgia 2007/1 genomes revealed multiple variable sites in 11 intergenic regions (IGR), including the tandem repeat sequence in IGR I73R/I329L (Supplementary Table S4). Among intergenic variable sites, the presence of a unique 19 bp (5′-TTGCAAACTAGATGTTTGA-3′) insertion in the IGR between EP424R and EP152R was the most remarkable difference compared to other full-length genome sequences available in the GenBank. Moreover, ASFV HK202103 presented 16 ORFs changes, including eight members of the MGF (MGF 360-1La, MGF 110-1L, MGF 110-3L, MGF 110-7L, MGF 110-13Lb, MGF 360-10L, MGF 505-4R, and MGF 505-9R), three genes encoding proteins involved in nucleotide metabolism (EP424R, NP419L, and D345L), a protein involved in viral morphogenesis (MGF 110-14L), and four proteins of unknown function (ASFV_G_ACD_00190, ASFV_G_ACD_00350, I267L, and DP60R) in comparison with Georgia 2007/1 strain (Supplementary Table S5).

### 3.5. Phylogenetic Analysis of HK202103 Sequence with Other ASFV Genotype II Complete Genomes

**Figure 5:**
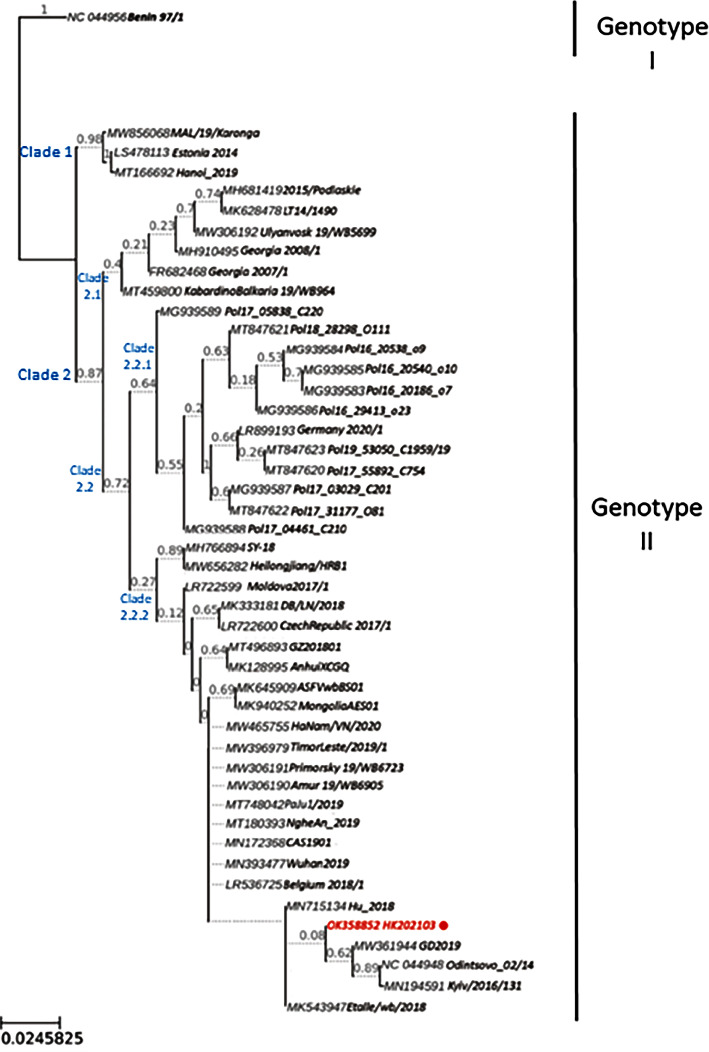
Phylogenetic analysis of the full-length genome of ASFV Hong Kong isolate (HK202103; GenBank accession: OK358852). The combined nucleotide alignment for 121 orthologs is used to build the tree. The branch length shows the nucleotide substitution rate. The bootstrap values from 1000 replicates are indicated on each node. The complete tree with all available full-length genome sequences from GenBank used for the analysis is provided in Supplementary Figure S3.

To determine the genetic relationship between ASFV HK202103 and other previously identified ASFVs, a phylogenetic tree was constructed by aligning the protein-coding sequences (CDS) of the HK202103 strain with 126 nonduplicated ASFV complete genomes obtained from GenBank (Supplementary Table S6). This phylogeny placed the HK202103 strain as a member of the genotype II ASFVs (Supplementary Figure S3). For the higher resolution of the phylogeny, the clade consisted of 45 genotype II ASFV strains, including HK202103, and an outgroup sequence from genotype I (isolate Benin 97/1 from the Republic of Benin) is presented in Figure 5. The genotype II strains were grouped into two clades, most of which were included in Clade 2. The strains belonging to Clade 2 were further divided into two clusters, Clade 2.1 and Clade 2.2. Clade 2.2 comprised a group of isolates from Poland (Clade 2.2.1) and another group of strains (Clade 2.2.2) that are the most recently circulating strains in Eastern Europe (Moldova2017/1, CzechRepublic 2017/1, Belgium 2018/1) and Asia, including China (AnhuiXCGQ, Wuhan2019, GZ201801, CAS1901), Vietnam (HaNam/VN/2020, NgheAn_2019), and South Korea (PaJu1/2019). In addition, several sequences isolated from wild boars were also included in Clade 2.2.2, such as those from Belgium (Etalle/wb/2018), Russia (Primorsky 19/WB6723, Amur 19/WB6905), and China (ASFVwbBS01). The HK202103 strain was most closely related to strains from Hungary (Hu_2018), Ukraine (Kyiv/2016/131), and China (GD2019) (Figure 5). Interestingly, despite HK202103 possessing identical p72 and EP402R gene sequences with ASFV SY-18 (Supplementary Figures S1 and S2), which was responsible for the first outbreak in China, the full-length genome of HK202103 was distantly related to ASFV SY-18 within the Clade 2.2.2., suggesting that the virus had experienced a highly adaptive evolution to the environment on other genes in a short generation time.

## 4. Discussion

Based on the epidemiological outbreak investigation, the virus might have been introduced to the farm on or after Dec 25, 2020, considering an estimated incubation period of 15 days for ASFV according to the Terrestrial Animal Health Code of the WOAH. Farm staff reported implementing enhanced biosecurity measures after noticing increased mortality from Jan 24, 2021. However, veterinary advice was only sought five days after (Jan 29, 2021), and the ASF diagnosis was made on Feb 3, with movement restrictions being implemented on the same day. This means there had been a minimum of ten days during which virus spread could have occurred within the farm and potentially from the farm to other susceptible domestic pigs and wild boar populations. Notably, the relatively small number of affected pigs at the beginning of the outbreak, their clinical presentation, and the postmortem results led the veterinary clinicians and pathologists to conclude that this slight increase in morbidity and mortality was not likely to have been caused by ASFV. ASF was, therefore, initially ranked very low on the list of differential diagnoses. The same diagnostic reasoning is likely applied in regions where ASFV is not endemic, particularly if other diseases with similar clinical or postmortem signs are endemic. This is an important observation since it compromises the ability to detect ASFV early. Nevertheless, the overall proportion of ASFV positives among the samples collected from different sheds and the corresponding transmission rate appeared to be low, and the extent of ASFV spread on the farm seemed limited. Several factors, including biosecurity measures implemented at the farm, may have reduced the risk of spread. For example, each farm worker was designated to work in specific pig sheds as a general farm management practice to prevent cross-contamination of infectious diseases between pigs kept in different sheds. When the disease outbreak was initially noticed by farm staff in the index shed in late Jan 2021, they isolated clinically affected pigs in a separate pen and culled around 40 pigs with clinical signs. These measures may have limited the spread of ASFV within the farm. Slow transmission of ASFV between animals has also been observed in outbreaks of domestic pig farms in other regions [37], where similar conditions in that early disease detection and immediate control measures were implemented to contain the virus, resulting in fewer animals becoming infected. It is also important to note that ASFV was not detected in other local pig farms, suggesting no onward spread occurred. Surveys to monitor suspicion of ASF were carried out in 42 other local pig farms, and the farmers were requested to submit nasal swab samples of sick and dead pigs for ASFV testing, of which all 866 samples tested negative. Overall, it can be concluded that the ASFV outbreak was contained with the control measures implemented on the outbreak farm.

**Figure 6:**
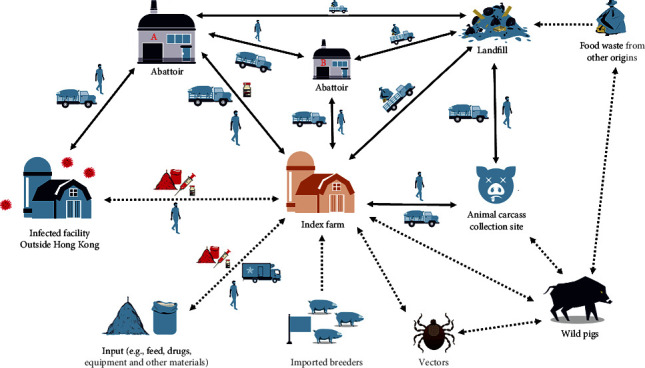
Potential risk factors and routes of ASFV introduction into the outbreak farm. High-risk and low-risk factors are indicated with solid and dashed lines, respectively. Adapted from Pfeiffer et al. [38]. Abattoir (A) main abattoir. Abattoir (B) minor abattoir.

Potential risk factors and routes of ASFV introduction into the farm, such as contaminated food or feed, personnel, live pigs, vehicles, and soft ticks are illustrated in Figure 6. Of the two possibilities associated with the exposure of pigs to contaminated pork products, only the one via leftovers from food consumed by farm workers was relevant since feeding food waste from pigs or pork origin is strictly banned in HK SAR. Concerning this, the farmer confirmed that the likelihood of farm workers bringing any pork products to the farm was low since the farm management provided food. Furthermore, the farm used mixed dry feed that was adequately stored for feeding the pigs. Although the possibility of the feed being contaminated with ASFV could not be ruled out, given the fact that while the same mixed feed was provided to all finishers, the outbreak pattern was initially sporadic, involving only a few finishers from a single shed rather than a spontaneous outbreak in finishers from different sheds; the feed was considered as an unlikely source for ASFV introduction. In addition, there was no direct evidence to show that the virus was introduced through contaminated feed since the ASFV genome was not detected in any tested feed ingredients. Similarly, it is unlikely that the virus was introduced via personnel from farms in Mainland China since COVID-19 travel restrictions limited cross-boundary movement between HK SAR and Mainland China for a year before the outbreak. However, inadequate track records of the farm on the movement of pigs, feed, vehicles, visitors, and other human activities weakened the source tracing. Moreover, no ticks were found in the environment or reported by farmers, while a small number of flies and rodents were observed inside the farm during the inspection. Although mechanical transmission of ASFV by rodents has not been characterized, rodents are considered low-risk vectors for ASF.

ASFV can be introduced into HK SAR via infected live pigs. An annual total of 792,000 live pigs (an average of 2,170 per day) were introduced daily from Mainland China in 2021, taken directly from the border to the abattoirs in HK SAR for slaughter [39]. The abattoir also slaughtered an average of 361 pigs per day from HK SAR during January 2021, which means there was an opportunity for indirect contact via vehicles and people [39]. From the first introduction of ASFV into Mainland China to the local farm incident, the virus was detected in imported pigs on three occasions at the city's main abattoir in 2019, indicating that abattoirs are potential routes of ASFV entry to HK SAR. To mitigate the risk of ASFV for local pig farms, strict biosecurity measures are implemented at the abattoirs, particularly through cleaning and disinfection of pig transport vehicles. Nevertheless, ASFV transmission risks may still exist for vehicles transporting pigs between pig farms and abattoirs, mainly when they go into some farms and carry the virus as fomites. Although pig transport vehicles had not entered the index farm area, the vehicles or drivers could still have taken pig excrement or other potentially contaminated materials near the farm and unintentionally brought in the virus, making it the most likely route of ASFV introduction for this outbreak, as depicted in Figure 6. In addition, waste collection vehicles removed animal waste stored in bins outside pig farms on a routine basis, while different vehicles picked up carcasses from animal carcass collection sites. These vehicles did not have access to farm areas where pigs were housed nor had any direct contact with abattoir wastes; thus, the risk of virus introduction via this pathway was considered negligible. Furthermore, there was no new introduction of live pigs or semen imported from the Mainland or overseas into this farm within the two years prior to this outbreak, which is considered unlikely as the source of ASFV introduction.

Furthermore, a routine ASF surveillance program for dead wild boars has been placed in HK SAR since Nov 1, 2019. Thirty-six wild boar carcasses in HK SAR tested negative for ASFV as of Jan 31, 2021. Thirty-two blood samples from live wild boars had also been collected during the same period via a conservation program and revealed ASFV-negative results, indicating no evidence of ASFV infection in wild boars in the region before this outbreak. While preparing the manuscript, ASFV was detected in wild boar carcasses found on Hong Kong Island (Sep 3, 2021, and Feb 28, 2022) and the northeastern and eastern region of the New Territories (Jan 14, 2022, and May 30, 2022). Although wild boars and feral pigs are possible sources of ASFV outbreaks as described in European outbreaks [40], this is considered to be unlikely for this case due to the relatively large geographical and temporal distance between the pig farm and the wild boar cases as well as the absence of wild boar intrusion as reported by the farmer and the physical barriers in place surrounding the farm. Further investigation is required to compare the full-length ASFV genomes recently circulating in Southern China with those of domestic pig and wild boar isolates identified in HK SAR to provide more insights into the genetic characterization and variation of ASFV in the region.

Lastly, the sequence described herein comprises the first ASFV full-length genome available in HK SAR. Several unique genetic variations in the ASFV HK202103 genome differ from other p72 genotype II strains. Thus, the role of genetic changes in the MGFs located at the 5′-end of the ASFV HK202103 variable region concerning its virulence phenotype remains to be determined. Although no specific mutations were found in genes involved in immune evasions, such as A238L, EP153L, EP402R, or MGF505-2R [41], several mutations in genes involved in nucleotide metabolism, transcription, and repair were found in HK202103 strain (Supplementary Table S4). In particular, the EP402R gene, responsible for the adsorption of red blood cells to virus-infected cells, has been considered an important genetic factor for ASFV attenuation [42–45]. Different from those recent naturally occurring mutants in Mainland China that exhibit lower virulence (e.g., HKJ/HRB1/20 strain (genotype II) or SD/DY-I/21 (genotype I)) in domestic pigs [22, 23], the EP402R gene of ASFV HK202103 was identical to the virulent ASFV-SY18 strain, which is responsible for the first Chinese ASF outbreak (Supplementary Figure S2). This was consistent with the HAD phenotype of HK202103 observed in the HAD assay, suggesting that CD2v was unaffected. Nevertheless, further studies are needed to examine the association between the genetic variability of the ASFV HK202103 genome and viral disease phenotype. Lastly, the nucleotide insertion in the intergenic region between EP424R and EP152R mentioned in the results was found in the genome of ASFV HK202103, which has not been detected in other genotype II ASFVs. Although a more comprehensive population study will be needed a priori, this variation could be used as a molecular marker for tracing disease spread in the future.

Overall, it can be concluded that in this particular outbreak, the spread of ASFV was slow and appeared to have spread from the index shed only to two other sheds. However, due to the small number of diagnostic samples collected from sheds other than the index shed, it cannot be excluded that further spread may have occurred. Notably, the relatively low morbidity and mortality of ASF on this farm resulted in delayed disease diagnosis preventing rapid implementation of control measures. In addition, the clinical presentation of ASF in terms of morbidity and mortality on this farm highlighted that it is essential for effective surveillance aimed at early detection for farmers, veterinarians, and pathologists to be educated about the different ways ASF can express itself in diverse domestic pig populations. Lastly, the definitive source of the virus was not identified, and likely possibilities included but were not limited to infected pigs imported for slaughter from Mainland China at the local abattoir and subsequent fomite transmission or contaminated materials brought into the farm.

## Figures and Tables

**Table 1:** Laboratory virology results of pigs tested ASFV positive in tissues or blood samples on a domestic pig farm in HK SAR.

Sampling date	Day	Pig	Building	Type	Sample type	rRT-PCR(Ct)	Gel PCR	HAD	VI
Jan 29, 2021	0	1	11	Finisher	Lymph node	20.5	+	+	+
					Spleen	18.2	+	+	+

Feb 3, 2021	5	2	11	Finisher	EDTA blood	17.8	+	ND	ND
		3	11	Finisher	EDTA blood	17.1	+	ND	ND
		4	11	Finisher	EDTA blood	15.3	+	+	+
		5	11	Finisher	EDTA blood	15.5	+	ND	ND
		6	11	Finisher	EDTA blood	21.9	+	ND	ND
		7	11	Finisher	EDTA blood	22.7	+	ND	ND

Feb 5, 2021	7	8	2	Sow	EDTA blood	16.3	+	ND	ND
		9	2	Sow	EDTA blood	17.0	+	+	+

Feb 11, 2021	13	10	12	Finisher	Lymph node	21.4	+	ND	ND
					Spleen	16.5	+	ND	ND

(+): positive; ND: not determined.

## Data Availability

The full-length genome sequence of ASFV HK202103 has been deposited in the GenBank database under the accession number OK358852.

## Supplementary Materials

Supplementary Materials Supplementary Table S1: molecular diagnostic results of various bacterial and viral diseases from tissues of the index pig. Supplementary Table S2: histopathological lesions on tissue samples collected from the dead finisher submitted to CityU VDL on Day 0. Supplementary Table S3: list of ASFV isolates with identical p72 nucleotide sequence to ASFV HK202103. Supplementary Table S4: summary of genetic variations detected in noncoding intergenic regions (IGR) of ASFV HK202103 genome compared to Georgia 2007/1. Supplementary Table S5: summary of genetic variations detected in coding regions of ASFV HK202103 genome compared to the reference Georgia 2007/1. Supplementary Table S6: ASFV full-length genome sequences used in this study. Supplementary Figure S1: phylogenetic tree constructed based on the aligned sequences of p72 complete gene derived from 53 nonredundant sequences available in GenBank. All identical sequences have been merged. The bootstrap values from 1000 replicates are indicated on each node. The HK202103 (OK358852) isolate is indicated in red (). Scale bar indicates the nucleotide substitution rate. The alignment result of full-length sequence is depicted on the right panel. Supplementary Figure S2: phylogenetic tree constructed based on the aligned sequences of CD2v complete gene derived from 62 nonredundant sequences available in GenBank. All identical sequences have been merged. The bootstrap values from 1000 replicates are indicated on each node. The HK202103 isolate (OK358852) is indicated in red (). Scale bar indicates the nucleotide substitution rate. The alignment result of full-length sequence is depicted on the right panel. Supplementary Figure S3: phylogenetic analysis of full-length genome of ASFV Hong Kong isolate (HK202103; GenBank accession: OK358852). The combined nucleotide alignment for 121 orthologs is used to build the tree. The branch length shows the nucleotide substitution rate. The bootstrap values from 1000 replicates are indicated on each node. The alignment result of full-length sequence is depicted on the right panel. The HK202103 isolate (OK358852) is indicated in red (). Scale bar indicates the nucleotide substitution rate.
