# Supplementary material for: Investigation of the First African Swine Fever Outbreak in a Domestic Pig Farm in Hong Kong
Source: Transbound Emerg Dis. 2023 May 18;2023:1720474. doi: 10.1155/2023/1720474 (PMC12017093; doi:10.1155/2023/1720474)
Supplement: Supplementary Materials — Supplementary Table S1: molecular diagnostic results of various bacterial and viral diseases from tissues of the index pig. Supplementary Table S2: histopathological lesions on tissue samples collected from the dead finisher submitted to CityU VDL on Day 0. Supplementary Table S3: list of ASFV isolates with identical p72 nucleotide sequence to ASFV HK202103. Supplementary Table S4: summary of genetic variations detected in noncoding intergenic regions (IGR) of ASFV HK202103 genome compared to Georgia 2007/1. Supplementary Table S5: summary of genetic variations detected in coding regions of ASFV HK202103 genome compared to the reference Georgia 2007/1. Supplementary Table S6: ASFV full-length genome sequences used in this study. Supplementary Figure S1: phylogenetic tree constructed based on the aligned sequences of p72 complete gene derived from 53 nonredundant sequences available in GenBank. All identical sequences have been merged. The bootstrap values from 1000 replicates are indicated on each node. The HK202103 (OK358852) isolate is indicated in red (). Scale bar indicates the nucleotide substitution rate. The alignment result of full-length sequence is depicted on the right panel. Supplementary Figure S2: phylogenetic tree constructed based on the aligned sequences of CD2v complete gene derived from 62 nonredundant sequences available in GenBank. All identical sequences have been merged. The bootstrap values from 1000 replicates are indicated on each node. The HK202103 isolate (OK358852) is indicated in red (). Scale bar indicates the nucleotide substitution rate. The alignment result of full-length sequence is depicted on the right panel. Supplementary Figure S3: phylogenetic analysis of full-length genome of ASFV Hong Kong isolate (HK202103; GenBank accession: OK358852). The combined nucleotide alignment for 121 orthologs is used to build the tree. The branch length shows the nucleotide substitution rate. The bootstrap values from 1000 replicates are indicat [file 1720474.f1.docx]

**Supplementary Materials**

| **Table S1.** Molecular diagnostics results of various bacterial and viral diseases from tissues of the index pig | | |
| --- | --- | --- |
| **Tissue** | **Pathogenic agent** | **Results** |
| Lung | *A. pleuropneumoniae* | Not detected |
|  | *Haemophilus parasuis* | Positive |
|  | *M. tuberculosis complex* | Not detected |
|  | *Mycobacterium avium* | Not detected |
|  | *Mycoplasma hyopneumoniae* | Not detected |
|  | Porcine Circovirus Type 2 | Not detected |
|  | PRRSV (EU) | Not detected |
|  | PRRSV (NA) | Not detected |
| Small intestine | *Clostridium perfringens* | Not detected |
|  | PEDV | Not detected |
|  | Porcine Circovirus Type 2 | Positive |
|  | Rotavirus (Genotypes A-G) | Not detected |
|  | TGEV | Not detected |
| Large intestine | Lawsonia intracellularis | Not detected |
| Synovium | Haemophilus parasuis | Positive |
| Meninges | Haemophilus parasuis | Positive |
| Kidney | Leptospira species | Not detected |
| Liver | Leptospira species | Not detected |
| Mediastinal lymph node | PRRSV (EU) | Not detected |
|  | PRRSV (NA) | Not detected |
| Sup inguinal lymph node | Porcine circovirus type 2 | Positive |

| **Table S2.** Histopathological lesions on tissue samples collected from the dead finisher submitted to CityU VDL on Day 0 | | | |
| --- | --- | --- | --- |
| Tissue | Histopathological Lesion | Chronicity | Severity |
| Brain | Multifocal, necrotizing neutrophilic, lymphohistiocytic vasculitis and meningoencephalitis with perivascular cuffs | Acute | Moderate to marked |
| Kidney | Multifocal fibrinonecrotizing glomerulonephritis and lymphoplasmacytic and eosinophilic, necrotizing interstitial nephritis | Acute | Moderate |
| Large intestine | Multifocal Peyer patches lymphoid necrosis | Acute | Moderate |
| Liver | Multifocal, random, necrotizing lymphoplasmacytic and eosinophilic hepatitis and vasculitis | Acute | Mild to moderate |
| Lung | Diffuse pulmonary edema and congestion | Acute | Moderate to marked |
|  | Multifocal fibrinonecrotic alveolitis and vasculitis | Acute | Moderate |
|  | Multifocal bronchus-associated lymphoid tissue necrosis | Acute | Mild |
| Pancreas | Multifocal necrosis with fibrin thrombi and haemorrhages | Acute | Moderate |
| Renal lymph node | Multifocal lymphoid necrosis and fibrinonecrotising vasculitis with haemorrhages | Acute | Moderate to marked |
| Skin | Multifocal, suppurative dermatitis with ischemic epidermis, fibrin thrombi and hemorrhages | Acute | Moderate |
| Small intestine | Multifocal Peyer patches lymphoid necrosis | Acute | Mild |
| Spinal cord | Multifocal, necrotizing neutrophilic, lymphohistiocytic vasculitis and myelitis with perivascular cuffs | Acute | Mild |
| Spleen | Multifocal lymphoid necrosis with fibrin thrombi and diffuse congestion | Acute | Marked |
| Synovium hock | Multifocal, fibrinonecrotic and lymphocytic, neutrophilic synovitis with fibrin thrombi | Acute | Mild |
| Tonsil | Multifocal necrosuppurative tonsillitis with intralesional cocci | Acute | Mild |
|  | Multifocal lymphoid necrosis and fibrinonecrotising vasculitis | Acute | Moderate |

| **Table S3.** List of ASFV isolates with identical p72 nucleotide sequence to ASFV HK202103 | | |
| --- | --- | --- |
| Number | GenBank Accession | Name |
| 1 | NC044948.1 | Odintsovo 02/14 |
| 2 | NC044959.2 | Georgia 2007/1 |
| 3 | FR682468.2 | Georgia 2007/1 |
| 4 | KJ195685.1 | Krasnodar 2012 |
| 5 | KP843857.1 | Odintsovo 02/14 |
| 6 | LR536725.1 | Belgium 2018/1 |
| 7 | LR722599.1 | Moldova 2017/1 |
| 8 | LR722600.1 | CzechRepublic 2017/1 |
| 9 | LR899193.1 | Germany 2020/1 |
| 10 | LS478113.1 | Estonia 2014 |
| 11 | MG939583.1 | Pol16 20186 o7 |
| 12 | MG939584.1 | Pol16 20538 o9 |
| 13 | MG939587.1 | Pol17 03029 C201 |
| 14 | MG939588.1 | Pol17 04461 C210 |
| 15 | MG939589.1 | Pol17 05838 C220 |
| 16 | MH681419.1 | ASFV/POL/2015/Podlaskie |
| 17 | MH713612.1 | ASFV-SY18 (major capsid protein p72 gene) |
| 18 | MH910495.1 | Georgia 2008/1 |
| 19 | MK128995.1 | AnhuiXCGQ |
| 20 | MK333180.1 | Pig/HLJ/2018 |
| 21 | MK333181.1 | DB/LN/2018 |
| 22 | MK543947.1 | Belgium/Etalle/wb/2018 |
| 23 | MK554698.1 | VNUA HY-ASF1 |
| 24 | MK628478.1 | ASFV/LT14/1490 |
| 25 | MK645909.1 | ASFV-wbBS01 |
| 26 | MK940252.1 | CN/2019/InnerMongolia-AES01 |
| 27 | MN172368.1 | ASFV/pig/China/CAS19-01/2019 |
| 28 | MN393476.1 | Wuhan 2019-1 |
| 29 | MN393477.1 | Wuhan 2019-2 |
| 30 | MN715134.1 | ASFV HU 2018 |
| 31 | MN793051.1 | VNUA/TB-ASF1 (major capsid protein p72 gene) |
| 32 | MT180393.1 | NgheAn 2019 |
| 33 | MT459800.1 | ASFV/Kabardino-Balkaria 19/WB-964 |
| 34 | MT496893.1 | GZ201801 |
| 35 | MT748042.1 | PaJu1/2019 |
| 36 | MT847620.1 | Pol17 55892 C754 |
| 37 | MT847621.1 | Pol18 28298 O111 |
| 38 | MT847622.1 | Pol17 31177 O81 |
| 39 | MT847623.2 | Pol19 53050 C1959/19 |
| 40 | MW306190.1 | ASFV/Amur 19/WB-6905 |
| 41 | MW306191.1 | ASFV/Primorsky 19/WB-6723 |
| 42 | MW306192.1 | ASFV/Ulyanovsk 19/WB-5699 |
| 43 | MW361944.1 | China/GD/2019 |
| 44 | MW396979.1 | ASFV/Timor-Leste/2019/1 |
| 45 | MW465755.1 | VNUA-ASFV-05L1/HaNam/VN/2020 |
| 46 | MW656282.1 | Pig/Heilongjiang/HRB1/2020 |
| 47 | MW856068.1 | MAL/19/Karonga |

| **Table S4**. Summary of genetic variations detected in non-coding intergenic regions (IGR) of ASFV HK202103 genome compared to Georgia 2007/1 | | | | |
| --- | --- | --- | --- | --- |
| **No.** | **Nucleotide position*** | **Intergenic region (IGR)** | **Type** | **Variation** |
| 1 | 1077-1078 | 5' repeat region | Insertion | 1 nt insertion |
| 2 | 1657-1658 | DP60R and ASFV G ACD 01990 | Insertion | 1 nt insertion |
|  | 1681-1682 |  | Insertion | 1 nt insertion |
|  | 1702-1704 |  | Deletion | 1 nt deletion |
| 3 | 2395-2594 | ASFV G ACD 01990 and MGF 360-1La | Substitution | 38 nt substitutions |
|  |  |  | Deletion | 279 nt deletions |
|  |  |  | Insertion | 3 nt insertion |
| 4 | 3683-3685 | MGF 360-1Lb and MGF 360-2L | Deletion | 1 nt deletion |
| 5 | 7504-7506 | MGF 360-3L and MGF 110-1L | Deletion | 1 nt deletion |
| 6 | 18565-18566 | MGF 360-4L and ASFV G ACD 00300 | Insertion | 1 nt insertion |
| 7 | 20522-20523 | ASFV G ACD 00320 and ASFV G ACD 00330 | Insertion | 3 nt insertion |
| 8 | 73837-73838 | EP424R and EP152R | Large insertion | 19 nt insertion |
| 9 | 174139-174140 | I73R and I329L | Large insertion | 10 nt insertion |
| 10 | 184513-184515 | I10L and L11L | Deletion | 1 nt deletion |
| 11 | 190281-190282 | ASFV G ACD 01980 and ASFV G ACD 01990 | Insertion | 1 nt insertion |
| *Nucleotide position based on ASFV HK202103 (GenBank OK358852) genome | | |  |  |

| Function type | Gene | Function | ASFV HK202103 (OK358852) | | | | Georgia 2007 (NC_044959) |
| --- | --- | --- | --- | --- | --- | --- | --- |
|  |  |  | Nucleotide position | Nucleotide change* | Amino acid change* | Effect on protein | Amino acid identity (%) |
| Multigene family | MGF 360-1La | 360 Multigene | 2,837-3,676 | Multiple | Multiple | Truncation | 86.5 |
|  | MGF 110-1L | 110 Multigene | 7,788 | C > T | W > stop | Truncation | 99.8 |
|  | MGF 110-3L | 110 Multigene | 9,055 | A > T | H > Q | Substitution | 99.2 |
|  | MGF 110-7L | 110 Multigene | 11418-11420 | Deletion C | Frameshift | Truncation | 48 |
|  | MGF 110-13Lb | 110 Multigene | 16390-16391 | Insertion 3xC | 1 aa insertion | Elongation | 100 |
|  | MGF 360-10L | 360 Multigene | 27,154 | T > C | N > S | Substitution | 99.7 |
|  | MGF 505-4R | 505 Multigene | 37,741 | T > C | None | None | 100 |
|  | MGF 505-9R | 505 Multigene | 45,305 | A > G | K > E | Substitution | 99.8 |
| Nucleotide metabolism, transcription, replication, and repair | EP424R | FTS J-like methyl transferase | 72,677 | C > G | T > S | Substitution | 99.8 |
|  | NP419L | DNA ligase | 135,262 | T > C | N > S | Substitution | 99.8 |
|  | D345L | lambda-like exonuclease | 146,929 | A > T | F > Y | Substitution | 99.7 |
|  |  |  | 147,219 | A > G | None | None |  |
| Viral morphogenesis | MGF 110-14L | 110 Multigene, XP124L | 14951-14954 | Deletion 2xC | Frameshift | Truncation | 100 |
| Unknown | ASFV_G_ACD_00190 | Unknown | 13297-13299 | Deletion A | Frameshift | Truncation | 25 |
|  | ASFV_G_ACD_00350 | Unknown | 20727-20731 | Deletion 3xG | 1 aa deletion | Truncation | 97.7 |
|  | I267L | Unknown | 171,610 | T > A | I > F | Substitution | 99.6 |
|  | DP60R | Unknown | 190868-190869 | Insertion A | Frameshift | Elongation | 77.8 |

**Table S5.** Summary of genetic variations detected in coding regions of ASFV HK202103 genome compared to the reference Georgia 2007/1

*Nucleotide and amino acid changes relative to the reference Georgia 2007/1 (GenBank NC_044959) genome

| **Table S6.** ASFV full-length genome sequences used in this study. | | | | | | |
| --- | --- | --- | --- | --- | --- | --- |
| **No.** | **GenBank No.** | **Name** | **Origin** | **Year** | **p72 genotype** | **References** |
| 1 | OK358852 | HK202103 | Hong Kong | 2021 | II | This study |
| 2 | LR722600.1 | CzechRepublic 2017/1 | Czech Rep. | 2017 | II | Forth et al. (2019) |
| 3 | LR722599.1 | Moldova 2017/1 | Moldova | 2017 | II | Forth et al. (2019) |
| 4 | LR536725.1 | Belgium 2018/1 | Belgium | 2018 | II | Forth et al. (2019) |
| 5 | LR899193.1 | Germany 2020/1 | Germany | 2020 | II | Sauter-Louis C et al. (2020) |
| 6 | LR899131.1 | Ken.rie1 | Germany | 2020 | II | Forth JH et al. (2020) |
| 7 | MN393477.1 | Wuhan 2019 | China | 2019 | II | Xiong D et al. (2020) |
| 8 | MN715134.1 | HU_2018 | Hungary | 2018 | II | Olasz F et al. (2019) |
| 9 | MZ202520.1 | K49 | Rep. of Congo | 1949 | I | Unpublished |
| 10 | MW396979.1 | TimorLeste/2019/1 | Timor Leste | 2019 | II | Mileto P et al (2021) |
| 11 | MK128995.1 | AnhuiXCGQ | China | 2018 | II | Bao, J et al. (2019) |
| 12 | LS478113.1 | Estonia 2014 | Estonia | 2014 | II | Zani L et al. (2018) |
| 13 | MW856068.1 | MAL/19/Karonga | Malawi | 2019 | II | Hakizimana, J.N et al. (2021) |
| 14 | MT459800.1 | KabardinoBalkaria 19/WB964 | Russia | 2019 | II | Mazloum, A et al. (2021) |
| 15 | MH766894.2 | ASFV SY-18 | China | 2018 | II | Miao, F. et al. (2018) |
| 16 | MN172368.1 | CAS1901 | China | 2019 | II | Jia, L et al. (2020) |
| 17 | MT496893.1 | GZ201801 | China | 2018 | II | Unpublished |
| 18 | MK645909.1 | ASFVwbBS01 | China | 2018 | II | Unpublished |
| 19 | NC_044957.1 | OURT 88/3 | Portugal | 1988 | I | Chapman, D.A et al. (2008) |
| 20 | FR_682468.2 | Georgia 2007/1 | Georgia | 2007 | II | Chapman, D.A et al. (2011) |
| 21 | MH681419.1 | 2015/Podlaskie | Poland | 2015 | II | Olesen et al. (2018) |
| 22 | MW800838.1 | Or_1984 | Italy | 1984 | I | Fiori, M.S et al. (2021) |
| 23 | MW736613.1 | 33747 WB | Italy | 2015 | I | Fiori, M.S et al. (2021) |
| 24 | MW736612.1 | 31208 | Italy | 2011 | I | Fiori, M.S et al. (2021) |
| 25 | MW736610.1 | 28928 | Italy | 2015 | I | Fiori, M.S et al. (2021) |
| 26 | MW736609.1 | 6396 WB | Italy | 2015 | I | Fiori, M.S et al. (2021) |
| 27 | MW736608.1 | 113049 WB | Italy | 2013 | I | Fiori, M.S et al. (2021) |
| 28 | MW736605.1 | 51268 | Italy | 2014 | I | Fiori, M.S et al. (2021) |
| 29 | MW736604.1 | 15998 | Italy | 2015 | I | Fiori, M.S et al. (2021) |
| 30 | MW736603.1 | 63525 WB | Italy | 2012 | I | Fiori, M.S et al. (2021) |
| 31 | MW736601.1 | 49179 WB | Italy | 2013 | I | Fiori, M.S et al. (2021) |
| 32 | MW736600.1 | 30322 | Italy | 2013 | I | Fiori, M.S et al. (2021) |
| 33 | MW736599.1 | 98039 | Italy | 2013 | I | Fiori, M.S et al. (2021) |
| 34 | MW736598.1 | 2019 WB | Italy | 2012 | I | Fiori, M.S et al. (2021) |
| 35 | MW736597.1 | 47039 | Italy | 2013 | I | Fiori, M.S et al. (2021) |
| 36 | MW723500.1 | 44076 | Italy | 2004 | I | Fiori, M.S et al. (2021) |
| 37 | MW723499.1 | 22137 | Italy | 2008 | I | Fiori, M.S et al. (2021) |
| 38 | MW723498.1 | 72912 WB | Italy | 2007 | I | Fiori, M.S et al. (2021) |
| 39 | MW723497.1 | 22649 | Italy | 2005 | I | Fiori, M.S et al. (2021) |
| 40 | MW723496.1 | 74377 | Italy | 2004 | I | Fiori, M.S et al. (2021) |
| 41 | MW723495.1 | 72398 WB | Italy | 2005 | I | Fiori, M.S et al. (2021) |
| 42 | MW723494.1 | 23221 | Italy | 2008 | I | Fiori, M.S et al. (2021) |
| 43 | MW723493.1 | 46830 | Italy | 2008 | I | Fiori, M.S et al. (2021) |
| 44 | MW723492.1 | 4996 WB | Italy | 2008 | I | Fiori, M.S et al. (2021) |
| 45 | MW723491.1 | Nu1995_4 | Italy | 1995 | I | Fiori, M.S et al. (2021) |
| 46 | MW723490.1 | Nu1995_3 | Italy | 1995 | I | Fiori, M.S et al. (2021) |
| 47 | MW723489.1 | Nu1995_2 | Italy | 1995 | I | Fiori, M.S et al. (2021) |
| 48 | MW723488.1 | Nu1993_2 | Italy | 1993 | I | Fiori, M.S et al. (2021) |
| 49 | MW723487.1 | Or1993_1 | Italy | 1993 | I | Fiori, M.S et al. (2021) |
| 50 | MW723486.1 | Nu1991_7 | Italy | 1991 | I | Fiori, M.S et al. (2021) |
| 51 | MW723485.1 | Nu1991_3 | Italy | 1991 | I | Fiori, M.S et al. (2021) |
| 52 | MW723484.1 | Nu1991_2 | Italy | 1991 | I | Fiori, M.S et al. (2021) |
| 53 | MW723483.1 | Nu1990_1 | Italy | 1990 | I | Fiori, M.S et al. (2021) |
| 54 | MW723482.1 | Nu1986 | Italy | 1986 | I | Fiori, M.S et al. (2021) |
| 55 | MW723481.1 | Nu1979 | Italy | 1979 | I | Fiori, M.S et al. (2021) |
| 56 | MW723480.1 | Ca1978_2 | Italy | 1978 | I | Fiori, M.S et al. (2021) |
| 57 | MN194591.1 | Kyiv/2016/131 | Ukraine | 2016 | II | Kovalenko, G et al. (2019) |
| 58 | MT748042.1 | PaJu1/2019 | Korea | 2019 | II | Nah, J. et al. (2020) |
| 59 | NC_044942.1 | BA71V | Spain | 1971 | I | Rodriguez, J.M et al. (2015) |
| 60 | NC_044956.1 | Benin 97/1 | Benin | 1997 | I | Chapman, D.A et al. (2008) |
| 61 | NC_044955.1 | 47/Ss/2008 | Italy | 2008 | I | Granberg et al. (2016) |
| 62 | NC_001659.2 | BA71V | Spain | 1971 | I | Yanez et al. (1995) |
| 63 | MN270980.1 | 22653/Ca/2014 | Italy | 2014 | I | Torresi, C et al. (2020) |
| 64 | MN270979.1 | 97/Ot/2012 | Italy | 2012 | I | Torresi, C et al. (2020) |
| 65 | MN270978.1 | 72407/Ss/2005 | Italy | 2005 | I | Torresi, C et al. (2020) |
| 66 | MN270977.1 | 26/Ss/2004 | Italy | 2004 | I | Torresi, C et al. (2020) |
| 67 | MN270976.1 | 60/Nu/1997 | Italy | 1997 | I | Torresi, C et al. (2020) |
| 68 | MN270975.1 | 142/Nu/1995 | Italy | 1995 | I | Torresi, C et al. (2020) |
| 69 | MN270974.1 | 141/Nu/1990 | Italy | 1990 | I | Torresi, C et al. (2020) |
| 70 | MN270973.1 | 85/Ca/1985 | Italy | 1985 | I | Torresi, C et al. (2020) |
| 71 | MN270972.1 | 140/Or/1985 | Italy | 1985 | I | Torresi, C et al. (2020) |
| 72 | MN270971.1 | 139/Nu/1981 | Italy | 1981 | I | Torresi, C et al. (2020) |
| 73 | MN270970.1 | 57/Ca/1979 | Italy | 1979 | I | Torresi, C et al. (2020) |
| 74 | MN270969.1 | 56/Ca/1978 | Italy | 1978 | I | Torresi, C et al. (2020) |
| 75 | MK940252.1 | MongoliaAES01 | China | 2019 | II | Unpublished |
| 76 | MK628478.1 | LT14/1490 | Lithuania | 2014 | II | Gallardo, C et al. (2017) |
| 77 | MK543947.1 | Etalle/wb/2018 | Belgium | 2018 | II | Gilliaux, G et al. (2019) |
| 78 | MW306192.1 | Ulyanovsk 19/WB5699 | Russia | 2019 | II | Mazloum, A et al. (2021) |
| 79 | MW306191.1 | Primorsky 19/WB6723 | Russia | 2019 | II | Mazloum, A et al. (2021) |
| 80 | MW306190.1 | Amur 19/WB6905 | Russia | 2019 | II | Mazloum, A et al. (2021) |
| 81 | MW465755.1 | HaNam/VN/2020 | Vietnam | 2020 | II | Truong, Q.L et al. (2021) |
| 82 | MN913970.1 | Liv13/33 (OmLF2) | France | 2017 | I | Chastagner, A et al. (2020) |
| 83 | MH025920.1 | R35/2015 | Uganda | 2015 | IX | Unpublished |
| 84 | MH025919.1 | N10/2015 | Uganda | 2015 | IX | Unpublished |
| 85 | MH025918.1 | R25/2015 | Uganda | 2015 | IX | Unpublished |
| 86 | MH025917.1 | R7/2015 | Uganda | 2015 | IX | Unpublished |
| 87 | MH025916.1 | R8/2015 | Uganda | 2015 | IX | Unpublished |
| 88 | MH910495.1 | Georgia 2008/1 | Georgia | 2008 | II | Farlow et al. (2008) |
| 89 | MW856067.1 | BUR/18/Rutana | Burundi | 2018 | X | Hakizimana, J.N et al. (2021) |
| 90 | MN641876.2 | RSA_W1_1999 | South Africa | 1999 | IV | Unpublished |
| 91 | MN641877.2 | RSA_2_2004 | South Africa | 2004 | XX | Unpublished |
| 92 | MN630494.2 | Zaire | Zaire | 1977 | XX | Unpublished |
| 93 | MT847623.2 | Pol19_53050_C1959/19 | Poland | 2017/2019 | II | Mazur-Panasiuk, N et al. (2020) |
| 94 | MT847622.1 | Pol17_31177_O81 | Poland | 2017/2019 | II | Mazur-Panasiuk, N et al. (2020) |
| 95 | MT847621.1 | Pol18_28298_O111 | Poland | 2017/2019 | II | Mazur-Panasiuk, N et al. (2020) |
| 96 | MT847620.1 | Pol17_55892_C754 | Poland | 2017/2019 | II | Mazur-Panasiuk, N et al. (2020) |
| 97 | MW656282.1 | Heilongjiang/HRB1 | China | 2020 | II | Sun, E. et al. (2021) |
| 98 | MW361944.1 | GD/2019 | China | 2019 | II | Unpublished |
| 99 | MT956648.1 | Uvira B53 | Congo | 2019 | X | Bisimwa, P.N et al. (2021) |
| 100 | MN336500.3 | RSA_2 | South Africa | 2008 | XXII | Ndlovu, S.S. et al. (2020) |
| 101 | MN318203.3 | LIV_5_40 | Zambia | 1983 | I | Ndlovu, S.S. et al. (2020) |
| 102 | MN394630.3 | SPEC_57 | South Africa | 1985 | VIII | Ndlovu, S.S. et al. (2020) |
| 103 | NC_044958.1 | strain E75 | Spain | 1975 | I | de Villiers et al. (2010) |
| 104 | NC_044947.1 | 26544/OG10 | Italy | 2010 | I | Bacciu, D. et al (2016) |
| 105 | NC_044946.1 | Ken06.Bus | Kenya | 2006 | IX | Bishop et al. (2015) |
| 106 | NC_044945.1 | Ken05/Tk1 | Kenya | 2005 | X | Bishop et al. (2015) |
| 107 | NC_044943.1 | NHV | Portugal | 1968 | I | Portugal et al. (2015) |
| 108 | NC_044941.1 | L60 | Portugal | 1960 | I | Portugal et al. (2015) |
| 109 | AY261363.1 | Pretoriuskop/96/4 | South Africa | 1996 | I | Unpublished |
| 110 | NC_044954.1 | Malawi Lil20/1/ tick | Malawi | 1983 | I | Haresnape and Wilkinson (1989) |
| 111 | NC_044953.1 | Mkuzi 1979 | South Africa | 1979 | I | Unpublished |
| 112 | NC_044951.1 | Tengani 62 | Malawi | 1962 | V/I | Pan (1992) |
| 113 | NC_044950.1 | Warmbaths | South Africa (ticks) | 2019 | I | Unpublished |
| 114 | NC_044949.1 | Warthog | Namibia | 2019 | I | Unpublished |
| 115 | NC_044948.1 | Odintsovo_02/14 | Russia | 2014 | II | Unpublished |
| 116 | NC_044944.1 | Kenya 1950 | Kenya | 1950 | X | Unpublished |
| 117 | MK333181.1 | DB/LN/2018 | China | 2018 | II | Wen, X. et al. (2019) |
| 118 | MG939589.1 | Pol17_05838_C220 | Poland | 2017 | II | Wozniakowski et al. (2018) |
| 119 | MG939588.1 | Pol17_04461_C210 | Poland | 2017 | II | Wozniakowski et al. (2018) |
| 120 | MG939587.1 | Pol17_03029_C201 | Poland | 2017 | II | Wozniakowski et al. (2018) |
| 121 | MG939586.1 | Pol16_29413_o23 | Poland | 2016 | II | Wozniakowski et al. (2018) |
| 122 | MG939585.1 | Pol16_20540_o10 | Poland | 2016 | II | Wozniakowski et al. (2018) |
| 123 | MG939584.1 | Pol16_20538_o9 | Poland | 2016 | II | Wozniakowski et al. (2018) |
| 124 | MG939583.1 | Pol16_20186_o7 | Poland | 2016 | II | Wozniakowski et al. (2018) |
| 125 | LR881473.1 | Arm/07/CBM/c4 | Arm/07/CBM/c | 2007 | I | Unpublished |
| 126 | MT180393.1 | NgheAn_2019 | Vietnam | 2019 | II | Unpublished |
| 127 | MT166692.1 | Hanoi_2019 | Vietnam | 2019 | II | Unpublished |


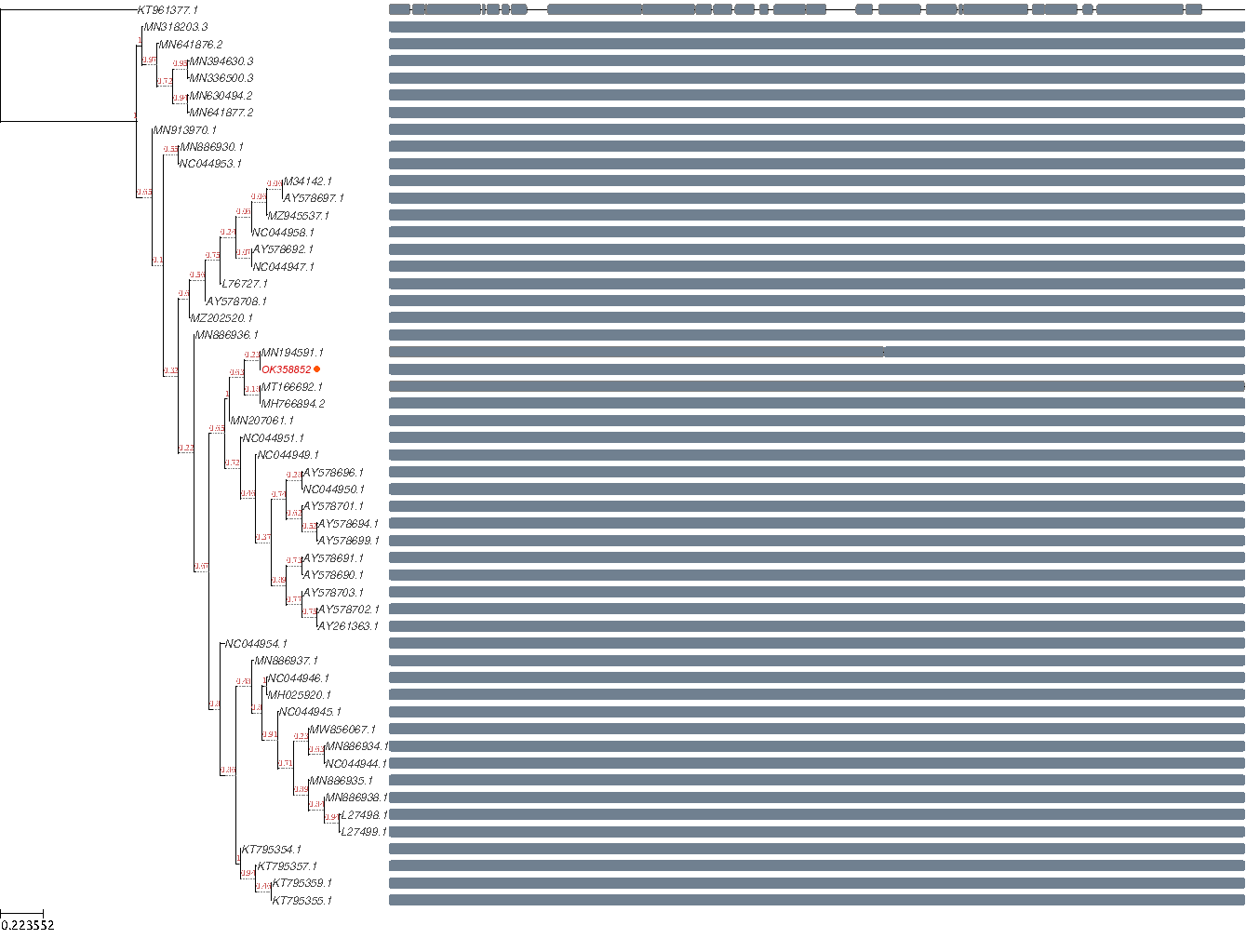


**Supplementary Figure S1.** Phylogenetic tree constructed based on the aligned sequences of p72 complete gene derived from 53 non-redundant sequences available in GenBank. All identical sequences have been merged. The bootstrap values from 1000 replicates are indicated on each node. The HK202103 (OK358852) isolate is indicated in red (•). Scale bar indicates nucleotide substitution rate. The alignment result of full-length sequence is depicted on the right panel.


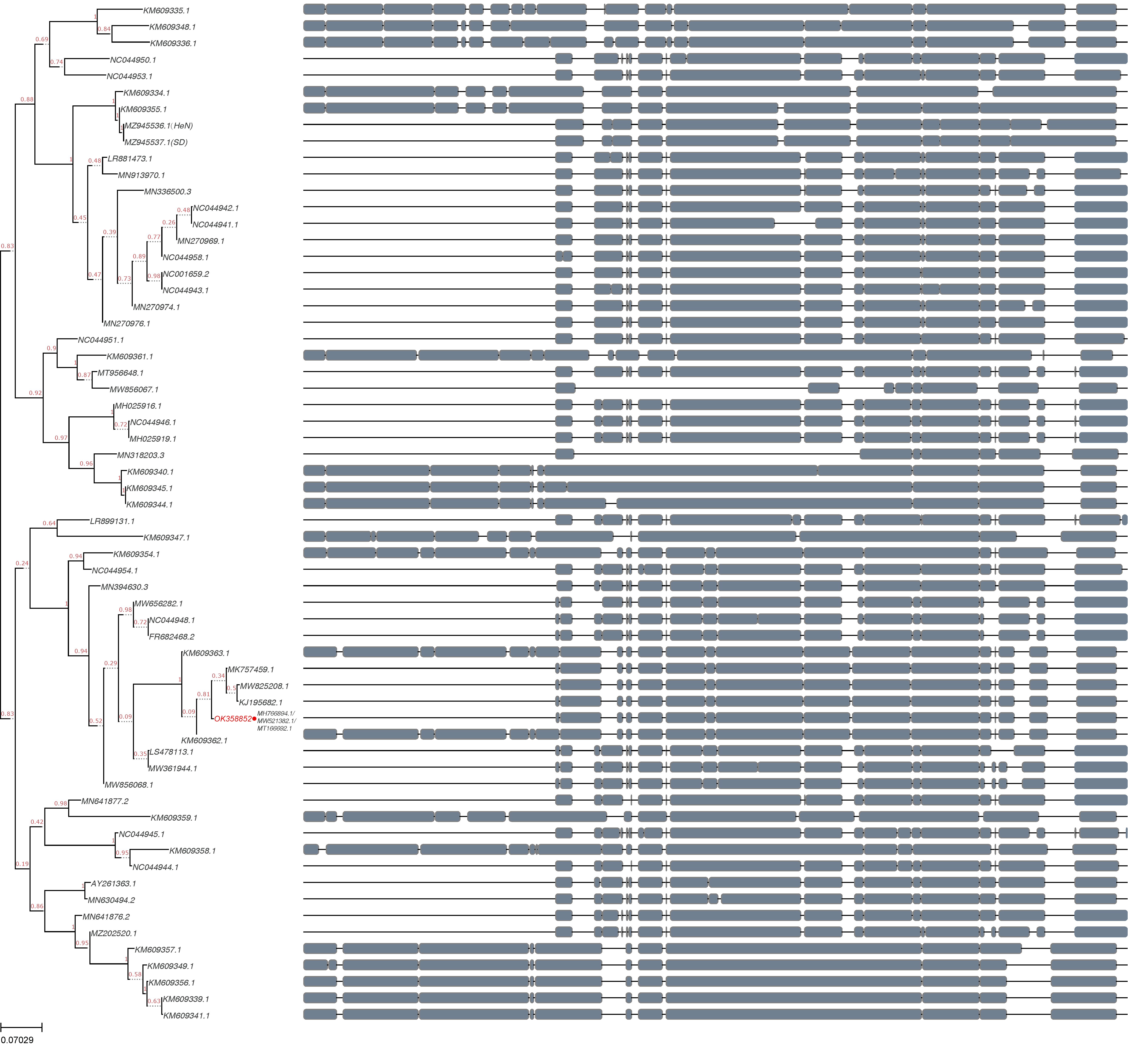


**Supplementary Figure S2.** Phylogenetic tree constructed based on the aligned sequences of CD2v complete gene derived from 62 non-redundant sequences available in GenBank. All identical sequences have been merged. The bootstrap values from 1000 replicates are indicated on each node. The HK202103 isolate (OK358852) is indicated in red (•). Scale bar indicates nucleotide substitution rate. The alignment result of full-length sequence is depicted on the right panel.

.
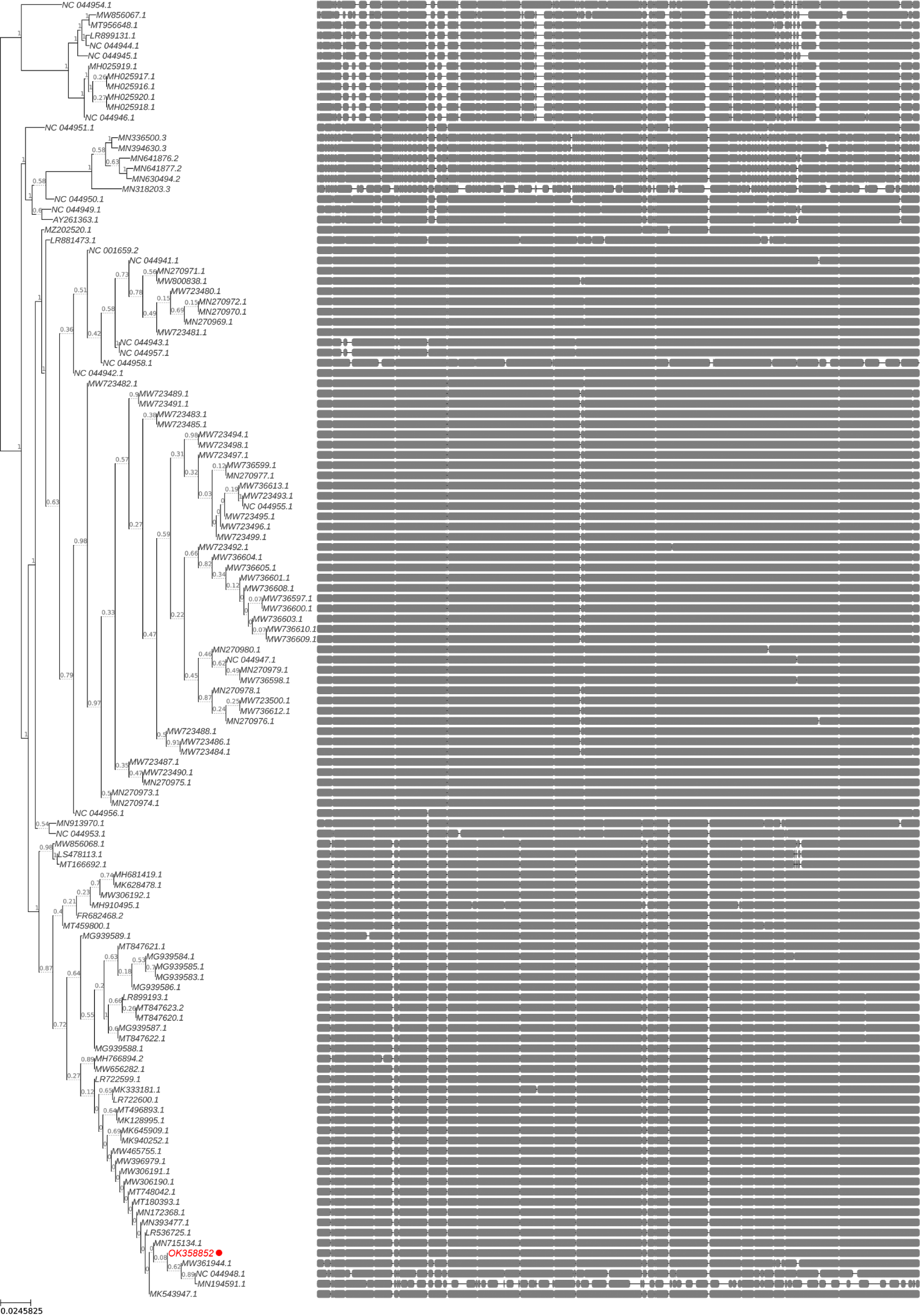


P72-based genotype II

**Supplementary Figure S3.** Phylogenetic analysis of full-length genome of ASFV Hong Kong isolate (HK202103; GenBank accession: OK358852). The combined nucleotide alignment for 121 orthologs are used to build the tree. The branch length shows the nucleotide substitution rate. The bootstrap values from 1000 replicates are indicated on each node. The alignment result of full-length sequence is depicted on the right panel. The HK202103 isolate (OK358852) is indicated in red (•). Scale bar indicates nucleotide substitution rate.
